# Supplementary material for: Mothers of children with major congenital anomalies have increased health care utilization over a 20-year post-birth time horizon
Source: PLoS One. 2021 Dec 8;16(12):e0260962. doi: 10.1371/journal.pone.0260962 (PMC8654179; doi:10.1371/journal.pone.0260962)
Supplement: S1 File — (DOCX) [file pone.0260962.s001.docx]

**Supplements**

**eAppendix 1.** Data Sources with Relevant References

**eAppendix 2.** STROBE Statement Checklist

**eAppendix 3.** Diagnostic Codes Used in the Study with Relevant References

**eAppendix 4.** List of Medical Specialists Included in the Primary Care Service Analyses

**eAppendix 5.** Healthcare Utilization by Affected Organ System: Period Prevalence Among Mothers Who Gave Birth to an Infant With a Major Congenital Anomalies Characteristics of Mothers (Exposed) and a Matched Comparison Cohort

**eAppendix 6.** Healthcare Utilization by Affected Organ System: Quantity of Services Among Mothers Who Gave Birth to an Infant With a Major Congenital Anomalies Characteristics of Mothers (Exposed) and a Matched Comparison Cohort

**eAppendix 7.** Healthcare Utilization by the Number of Child Hospitalizations within the First Year: Period Prevalence Among Mothers Who Gave Birth to an Infant With a Major Congenital Anomalies Characteristics of Mothers (Exposed) and a Matched Comparison Cohort

**eAppendix 8.** Healthcare Utilization by the Number of Child Hospitalizations within the First Year: Quantity of Services Among Mothers Who Gave Birth to an Infant With a Major Congenital Anomalies Characteristics of Mothers (Exposed) and a Matched Comparison Cohort

**eAppendix 9.** Healthcare Utilization by Income: Period Prevalence Among Mothers Who Gave Birth to an Infant with a Major Congenital Anomalies (Exposed Cohort) and a Matched Comparison Cohort

**eAppendix 10.** Healthcare Utilization by Income: Quantity of Services Among Mothers Who Gave Birth to an Infant with a Major Congenital Anomalies (Exposed Cohort) and a Matched Comparison Cohort

**eAppendix 11.** Healthcare Utilization by MCA-related history: Period Prevalence Among Mothers Who Gave Birth to an Infant with a Major Congenital Anomalies (Exposed Cohort) and a Matched Comparison Cohort

**eAppendix 12.** Healthcare Utilization by MCA-related history: Quantity of Services Among Mothers Who Gave Birth to an Infant with a Major Congenital Anomalies (Exposed Cohort) and a Matched Comparison Cohort

**eAppendix 13.** Healthcare Utilization by the Modified Charlson Comorbidity Index (CCI) Score: Period Prevalence Among Mothers Who Gave Birth to an Infant with a Major Congenital Anomalies (Exposed Cohort) and a Matched Comparison Cohort

**eAppendix 14.** Healthcare Utilization by the Modified Charlson Comorbidity Index (CCI) Score: Quantity of Services Among Mothers Who Gave Birth to an Infant with a Major Congenital Anomalies (Exposed Cohort) and a Matched Comparison Cohort

**eAppendix 1.** Data Sources with Relevant References

We used the following data sources. The Danish Medical Birth Registry[[1]](https://paperpile.com/c/ngVCz8/vE3p), Danish Civil Registration System[[2]](https://paperpile.com/c/ngVCz8/nplB), the Danish National Patient Registry, the Danish Psychiatric Central Research Register[[3]](https://paperpile.com/c/ngVCz8/nmkE), and the Danish National Health Service Register[[4]](https://paperpile.com/c/ngVCz8/CcnF).

References

1 [Bliddal M, Broe A, Pottegård A, *et al.* The Danish Medical Birth Register. *Eur J Epidemiol* 2018;**33**:27–36.](http://paperpile.com/b/ngVCz8/vE3p)

2 [Schmidt M, Pedersen L, Sørensen HT. The Danish Civil Registration System as a tool in epidemiology. *Eur J Epidemiol* 2014;**29**:541–9.](http://paperpile.com/b/ngVCz8/nplB)

3 [Mors O, Perto GP, Mortensen PB. The Danish Psychiatric Central Research Register. *Scand J Public Health* 2011;**39**:54–7.](http://paperpile.com/b/ngVCz8/nmkE)

4 [Andersen JS, Olivarius NDF, Krasnik A. The Danish National Health Service Register. *Scand J Public Health* 2011;**39**:34–7.](http://paperpile.com/b/ngVCz8/CcnF)

**eAppendix 2.** STROBE Statement Checklist

The study followed the Strengthening the Reporting of Observational Studies in Epidemiology (STROBE) reporting guidelines. Details are provided below.

|  | **Item No** | **Recommendation** |  | **Page** |
| --- | --- | --- | --- | --- |
| **Title and abstract** | 1 | (*a*) Indicate the study’s design with a commonly used term in the title or the abstract |  | 1 |
|  |  | (*b*) Provide in the abstract an informative and balanced summary of what was done and what was found |  | 2 |
| **Introduction** | | |  |  |
| Background/rationale | 2 | Explain the scientific background and rationale for the investigation being reported |  | 4 |
| Objectives | 3 | State specific objectives, including any prespecified hypotheses |  | 4 |
| **Methods** | | |  |  |
| Study design | 4 | Present key elements of study design early in the paper |  | 4-5 |
| Setting | 5 | Describe the setting, locations, and relevant dates, including periods of recruitment, exposure, follow-up, and data collection |  | 5 |
| Participants | 6 | (*a*) Give the eligibility criteria, and the sources and methods of selection of participants. Describe methods of follow-up |  | 5 |
|  |  | (*b*) For matched studies, give matching criteria and number of exposed and unexposed |  | 5 |
| Variables | 7 | Clearly define all outcomes, exposures, predictors, potential confounders, and effect modifiers. Give diagnostic criteria, if applicable |  | 5-7 |
| Data sources/ measurement | 8* | For each variable of interest, give sources of data and details of methods of assessment (measurement). Describe comparability of assessment methods if there is more than one group |  | 5 |
| Bias | 9 | Describe any efforts to address potential sources of bias |  | 15 |
| Study size | 10 | Explain how the study size was arrived at |  | n/a |
| Quantitative variables | 11 | Explain how quantitative variables were handled in the analyses. If applicable, describe which groupings were chosen and why |  | 5-6 |
| Statistical methods | 12 | (*a*) Describe all statistical methods, including those used to control for confounding |  | 7 |
|  |  | (*b*) Describe any methods used to examine subgroups and interactions |  | 7-8 |
|  |  | (*c*) Explain how missing data were addressed |  |  |
|  |  | (*d*) If applicable, explain how loss to follow-up was addressed |  |  |
|  |  | (*e*) Describe any sensitivity analyses |  | 8 |
| **Results** | | |  |  |
| Participants | 13* | (a) Report numbers of individuals at each stage of study—eg numbers potentially eligible, examined for eligibility, confirmed eligible, included in the study, completing follow-up, and analysed |  | Figure 1 |
|  |  | (b) Give reasons for non-participation at each stage |  | Figure 1 |
|  |  | (c) Consider use of a flow diagram |  | Figure 1 |
| Descriptive data | 14* | (a) Give characteristics of study participants (eg demographic, clinical, social) and information on exposures and potential confounders |  | 8 |
|  |  | (b) Indicate number of participants with missing data for each variable of interest |  | Figure 1 |
|  |  | (c) Summarise follow-up time (eg, average and total amount) |  | Table 1 |
| Outcome data | 15* | Report numbers of outcome events or summary measures over time |  | Figures  2 & 4 |
| Main results | 16 | (*a*) Give unadjusted estimates and, if applicable, confounder-adjusted estimates and their precision (eg, 95% confidence interval). Make clear which confounders were adjusted for and why they were included |  | Figures 2 & 4 |
|  |  | (*b*) Report category boundaries when continuous variables were categorized |  |  |
|  |  | (*c*) If relevant, consider translating estimates of relative risk into absolute risk for a meaningful time period |  |  |
| Other analyses | 17 | Report other analyses done—eg analyses of subgroups and interactions, and sensitivity analyses |  | 11 |
| **Discussion** | | |  |  |
| Key results | 18 | Summarise key results with reference to study objectives |  | 12 |
| Limitations | 19 | Discuss limitations of the study, taking into account sources of potential bias or imprecision. Discuss both direction and magnitude of any potential bias |  | 15 |
| Interpretation | 20 | Give a cautious overall interpretation of results considering objectives, limitations, multiplicity of analyses, results from similar studies, and other relevant evidence |  | 13-15 |
| Generalisability | 21 | Discuss the generalisability (external validity) of the study results |  | 15 |
| **Other information** | | |  |  |
| Funding | 22 | Give the source of funding and the role of the funders for the present study and, if applicable, for the original study on which the present article is based |  |  |

*Give information separately for exposed and unexposed groups.

**Note:** An Explanation and Elaboration article discusses each checklist item and gives methodological background and published examples of transparent reporting. The STROBE checklist is best used in conjunction with this article (freely available on the Web sites of PLoS Medicine at http://www.plosmedicine.org/, Annals of Internal Medicine at http://www.annals.org/, and Epidemiology at http://www.epidem.com/). Information on the STROBE Initiative is available at <http://www.strobe-statement.org>.

**eAppendix 3.** Diagnostic Codes Used in the Study with Relevant References

1. EXPOSURE

Major congenital anomalies by organ system (based on EUROCAT categorization of subgroups of congenital anomalies)

|  | ICD 10 |
| --- | --- |
| All congenital anomalies | All Q chapter *excluding* the following minor congenital anomalies  Exclusions: Q671, Q674, Q672, Q189, Q670, Q673, Q753, Q135, Q101, Q102, Q752, Q103, Q105,Q170, Q171, Q172, Q173, Q174, Q175, Q179, Q180, Q181, Q182, Q189, Q385, Q186, Q382, Q184, Q187, Q185, Q674, Q381, Q189, Q680, Q740G, Q845, Q8280, Q653-656, Q668, Q669, Q665, Q845, Q663, Q666, Q662, Q667, Q664, Q833, Q825, Q766, Q765, Q683, Q684, Q685, Q675, Q764L, Q676, Q682A, Q677, Q678, Q760, Q767C, Q270, Q250 (if GA < 37 weeks), Q211C, Q256 (if GA < 37 weeks), Q261, Q254E, Q331, Q314, Q315, Q320, Q401, Q430, Q400, Q633, Q610, Q627, Q552F, Q527, Q525, Q523, Q552, Q53, Q899, Q950, Q951  Q53 and Q65 specifically excluded due to poor validity in Danish National Patient Register  Also included: D215 (benign neoplasm of pelvis), D821 (DiGeorge Syndrome), D1810 (lymphangioma), P350, P351, P371 |
| Nervous system | Q00-Q07 |
| Eye | Q10-Q15 (exclusions: Q101-Q103, Q105, Q135) |
| Ear, face and neck | Q16-Q18 (exclusions: Q170-Q175, Q179, Q180-Q182, Q184-Q187, Q189) |
| Congenital heart disease | Q20-Q26 (exclusions: Q250 (if GA < 37 weeks), Q211C, Q256 (if GA < 37 weeks), Q254E, Q261 |
| Respiratory | Q30-34 (exclusions: Q314, Q315, Q320, Q331) |
| Oro-facial cleft | Q35-Q37 |
| Digestive system | Q38-Q45, Q790 (exclusions: Q381, Q382, Q38  5, Q400, Q401, Q430) |
| Abdominal wall defects | Q792, Q793, Q795 |
| Urinary | Q60-Q64, Q794 (exclusions: Q610, Q627, Q633) |
| Other exclusions | Q760, Q764L, Q765, Q766, Q767C, Q825, Q8280, Q833, Q899, Q950, Q951, Q845 |
| Other anomalies/syndromes | As below |
| Skeletal dysplasia | Q740B, Q77, Q780, Q782-Q788 |
| Craniosynostosis | Q750 |
| Congenital constriction bands/amniotic bands | No code |
| Situs inversus | Q893 |
| Conjoined twins | Q894 |
| Congenital skin disorders | Q80-Q82 |
| VATER/VACTERL syndrome | Q872G |
| Vascular disruption anomalies | Q411, Q412, Q418, Q710, Q712, Q713, Q720, Q722, Q723, Q730, Q793, Q795, Q798S, Q870G |
| Teratogenic syndromes with malformations | Q86, P350, P351, P371 |
| Fetal alcohol syndrome  Valproate syndrome | Q860  No code |
| Maternal infections resulting in malformations | P350, P351, P371 |
| Genetic syndromes and microdeletions | Q447B, Q619A, Q751, Q754, Q87, Q936, D821 |
| Chromosomal | Q90-Q92; Q93, Q96-99 |

Note: At least 1 code in two or more organ systems required for inclusion as multiorgan major congenital anomalies.

2. COVARIATES

(1) Diabetes Mellitus

| **Type** | **ICD 10** |
| --- | --- |
| 1 and 2 | O240, O241 |
| Gestational | O244, O249 |
| Unspecified | E12, E13, E14, O242, O243 |

(2) Modified Charlson Comorbidity Index

| **Conditions and Weight** | | **ICD 10** |
| --- | --- | --- |
| Myocardial infarction | Weight 1 | I21;I22;I23 |
| Congestive heart failure | Weight 1 | I50; I11.0; I13.0; I13.2 |
| Peripheral vascular disease | Weight 1 | I70; I71; I72; I73; I74; I77 |
| Cerebrovascular disease | Weight 1 | I60-I69; G45; G46 |
| Dementia | Weight 1 | F00-F03; F05.1; G30 |
| Chronic pulmonary disease | Weight 1 | J40-J47; J60-J67; J68.4; J70.1; J70.3; J84.1; J92.0; J96.1; J98.2; J98.3 |
| Connective tissue disease | Weight 1 | M05; M06; M08; M09; M30; M31; M32; M33; M34; M35; M36; D86 |
| Ulcer disease | Weight 1 | K22.1; K25-K28 |
| Mild liver disease | Weight 1 | B18; K70.9; K71; K73; K74; K76.0 |
| Diabetes without end-organ damage | Weight 1 | E10.0, E10.1; E10.9; E11.0; E11.1; E11.9 |
| Diabetes with end-organ damage | Weight 2 | E10.2-E10.8, E11.2-E11.8 |
| Hemiplegia | Weight 2 | G81; G82 |
| Moderate to severe renal disease | Weight 2 | I12; I13; N00-N05; N07; N11; N14; N17-N19; Q61 |
| Non-metastatic solid tumour | Weight 2 | C00-C75 |
| Leukaemia | Weight 2 | C91-C95 |
| Lymphoma | Weight 2 | C81-C85; C88; C90; C96 |
| Moderate to severe liver disease | Weight 3 | B15.0; B16.0; B16.2; B19.0; K72; K76.6; I85 |
| Metastatic cancer | Weight 6 | C76-C80 |
| AIDS | Weight 2 | B21-B24 |

Note: ICD-10 codes K70.0-K70.3 (mild liver disease) and K70.4 (moderate to severe liver disease) codes removed as these are liver diseases associated with alcohol-related disease.

Charlson codes removed (separate covariates):

a) Diabetes without end-organ damage (ICD10: E10.0, E10.1; E10.9; E11.0; E11.1; E11.9)

b) Diabetes with end-organ damage (ICD10: E10.2-E10.8, E11.2-E11.8)

c) Liver disease associated with alcohol use [K70.0-K70.3 (mild liver disease) and K70.4 (moderate to severe liver disease)].

(3) Chronic hypertension

| **ICD 10** |
| --- |
| I10-I15, I674 |

(4) Alcohol-related diseases

| **ICD 10** |
| --- |
| G312, G621, G721, I426, K292, K860, R780, Z721, F10, T51, K70 |

(5) Spontaneous Abortion

| **ICD 10** | |
| --- | --- |
| Missed Abortion | O021, O021A |
| Spontaneous Abortion | O03x |

(6) Pregnancy complications

| **ICD 10** | |
| --- | --- |
| A) Placental | |
| Pre-eclampsia | O140—O142, O149, O150-O159 |
| Gestational or unspecified Hypertension | O13, O16 |
| Placental abruption | O45 |
| Placental infarction | O43.1, O43.8, O43.9 |
|  |  |
| B) Non-Placental | |
| Intrauterine hypoxia and birth asphyxia | P20, P21 |
| Uterine rupture | O71.0, O71.1 |
| Umbilical cord prolapse or vasa previa | O69 |
| Amniotic fluid embolism | O88.1 |
| Fetal-maternal hemorrhage | O43.0 |
| Chorioamnionitis | O41.1 |

Other notes on covariates:

Marital status was ascertained from Danish Civil Registry at the time of the index birth. A six-month allowance was made post-index birth to allow for delayed registration of marriage.

3. OUTCOMES

(1) Inpatient care codes used in the study

|  | **ICD 10** |
| --- | --- |
| Hospital contacts | All entries in the Danish National Patient Registry |

(2) Psychiatric care codes used in the study

|  | **ICD 10** |
| --- | --- |
| All psychiatric diagnoses | F00-F99 |

**eAppendix 4.** List of Medical Specialists Included in the Primary Care Service Analyses

- General practitioners
- General medical care
- Internal medicine
- Ear/Nose/Throat - Ophthalmologist Assistance, Ear medical care
- Dental care
- Specialty practitioners
  - Anesthesiology
  - Dermato-venereology
  - Rheumatology
  - Gynecology/Obstetrics
  - Neuromedicine
  - Physiotherapy
  - Chiropractic
  - Free physiotherapy
  - Chiropractic chroniclers
  - Physiotherapy

**eAppendix 5.** Healthcare Utilization by Affected Organ System: Period Prevalence Among Mothers Who Gave Birth to an Infant with a Major Congenital Anomalies (Exposed Cohort) and a Matched Comparison Cohort

|  | Adjusted Risk Ratio (95% CI) |
| --- | --- |
| Primary Care Utilization |  |
| 0-6 years after birth |  |
| Single-organ MCA | 1.00 (1.00–1.00) |
| Multiple-organ MCAs | 1.00 (1.00–1.00) |
| 7-13 years after birth |  |
| Single-organ MCA | 1.00 (1.00–1.00) |
| Multiple-organ MCAs | 1.00 (1.00–1.00) |
| 14-18 years after birth |  |
| Single-organ MCA | 1.00 (1.00–1.00) |
| Multiple-organ MCAs | 1.00 (1.00–1.00) |
| Outpatient Clinic Utilization |  |
| 0-6 years after birth |  |
| Single-organ MCA | 1.01 (1.01–1.02) |
| Multiple-organ MCAs | 1.04 (1.02–1.05) |
| 7-13 years after birth |  |
| Single-organ MCA | 1.01 (1.00–1.02) |
| Multiple-organ MCAs | 1.05 (1.02–1.07) |
| 14-18 years after birth |  |
| Single-organ MCA | 1.02 (1.01–1.04) |
| Multiple-organ MCAs | 1.03 (1.00–1.07) |
| Inpatient Care Utilization |  |
| 0-6 years after birth |  |
| Single-organ MCA | 1.12 (1.11–1.13) |
| Multiple-organ MCAs | 1.21 (1.18–1.24) |
| 7-13 years after birth |  |
| Single-organ MCA | 1.07 (1.04–1.09) |
| Multiple-organ MCAs | 1.13 (1.07–1.19) |
| 14-18 years after birth |  |
| Single-organ MCA | 1.08 (1.03–1.13) |
| Multiple-organ MCAs | 1.16 (1.03–1.30) |
| Surgical Care Utilization |  |
| 0-6 years after birth |  |
| Single-organ MCA | 1.05 (1.04–1.06) |
| Multiple-organ MCAs | 1.15 (1.12–1.19) |
| 7-13 years after birth |  |
| Single-organ MCA | 1.03 (1.01–1.05) |
| Multiple-organ MCAs | 1.10 (1.04–1.15) |
| 14-18 years after birth |  |
| Single-organ MCA | 1.04 (1.00–1.08) |
| Multiple-organ MCAs | 1.12 (1.02–1.23) |
| Psychiatric Clinic Utilization |  |
| 0-6 years after birth |  |
| Single-organ MCA | 1.14 (1.06–1.21) |
| Multiple-organ MCAs | 1.14 (0.94–1.39) |
| 7-13 years after birth |  |
| Single-organ MCA | 1.11 (1.02–1.21) |
| Multiple-organ MCAs | 1.15 (0.93–1.42) |
| 14-18 years after birth |  |
| Single-organ MCA | 1.13 (0.98–1.30) |
| Multiple-organ MCAs | inestimable |
| Psychiatric Inpatient Care Utilization |  |
| 0-6 years after birth |  |
| Single-organ MCA | 1.03 (0.90–1.18) |
| Multiple-organ MCAs | 1.20 (0.84–1.70) |
| 7-13 years after birth |  |
| Single-organ MCA | 1.20 (1.04–1.39) |
| Multiple-organ MCAs | inestimable |
| 14-18 years after birth |  |
| Single-organ MCA | inestimable |
| Multiple-organ MCAs | inestimable |

**eAppendix 6.** Healthcare Utilization by Affected Organ System: Quantity of Services Among Mothers Who Gave Birth to an Infant with a Major Congenital Anomalies (Exposed Cohort) and a Matched Comparison Cohort

|  | Adjusted Rate Ratio (95% CI) |
| --- | --- |
| Primary Care Utilization |  |
| 0-6 years after birth |  |
| Single-organ MCA | 1.05 (1.04–1.06) |
| Multiple-organ MCAs | 1.03 (1.00–1.06) |
| 7-13 years after birth |  |
| Single-organ MCA | 1.05 (1.03–1.06) |
| Multiple-organ MCAs | 1.04 (1.00–1.09) |
| 14-18 years after birth |  |
| Single-organ MCA | 1.07 (1.04–1.10) |
| Multiple-organ MCAs | 1.02 (0.94–1.10) |
| Outpatient Clinic Utilization |  |
| 0-6 years after birth |  |
| Single-organ MCA | 1.18 (1.15–1.20) |
| Multiple-organ MCAs | 1.36 (1.29–1.42) |
| 7-13 years after birth |  |
| Single-organ MCA | 1.13 (1.09–1.16) |
| Multiple-organ MCAs | 1.13 (1.05–1.22) |
| 14-18 years after birth |  |
| Single-organ MCA | 1.11 (1.06–1.17) |
| Multiple-organ MCAs | 1.09 (0.96–1.24) |
| Inpatient Care Utilization |  |
| 0-6 years after birth |  |
| Single-organ MCA | 1.35 (1.32–1.37) |
| Multiple-organ MCAs | 1.77 (1.68–1.87) |
| 7-13 years after birth |  |
| Single-organ MCA | 1.13 (1.09–1.18) |
| Multiple-organ MCAs | 1.22 (1.11–1.34) |
| 14-18 years after birth |  |
| Single-organ MCA | 1.12 (1.03–1.22) |
| Multiple-organ MCAs | 1.20 (0.99–1.44) |
| Surgical Care Utilization |  |
| 0-6 years after birth |  |
| Single-organ MCA | 1.13 (1.10–1.15) |
| Multiple-organ MCAs | 1.33 (1.26–1.41) |
| 7-13 years after birth |  |
| Single-organ MCA | 1.10 (1.06–1.14) |
| Multiple-organ MCAs | 1.13 (1.03–1.23) |
| 14-18 years after birth |  |
| Single-organ MCA | 1.08 (1.02–1.15) |
| Multiple-organ MCAs | 1.15 (0.98–1.35) |
| Psychiatric Clinic Utilization |  |
| 0-6 years after birth |  |
| Single-organ MCA | 1.10 (0.94–1.29) |
| Multiple-organ MCAs | 0.63 (0.44–0.91) |
| 7-13 years after birth |  |
| Single-organ MCA | 1.18 (0.98–1.42) |
| Multiple-organ MCAs | 1.46 (0.94–2.27) |
| 14-18 years after birth |  |
| Single-organ MCA | 1.21 (0.89–1.63) |
| Multiple-organ MCAs | inestimable |
| Psychiatric Inpatient Care Utilization |  |
| 0-6 years after birth |  |
| Single-organ MCA | 1.07 (0.87–1.32) |
| Multiple-organ MCAs | 1.51 (0.76–3.01) |
| 7-13 years after birth |  |
| Single-organ MCA | 1.23 (0.92–1.65) |
| Multiple-organ MCAs | inestimable |
| 14-18 years after birth |  |
| Single-organ MCA | inestimable |
| Multiple-organ MCAs | inestimable |

**eAppendix 7.** Healthcare Utilization by the Number of Child Hospitalizations within the First Year: Period Prevalence Among Mothers Who Gave Birth to an Infant with a Major Congenital Anomalies (Exposed Cohort) and a Matched Comparison Cohort

|  | Adjusted Risk Ratio (95% CI) |
| --- | --- |
| Primary Care Utilization |  |
| 0-6 years after birth |  |
| 0 hospitalization | 1.00 (1.00–1.00) |
| 1-3 hospitalization | 1.00 (1.00–1.00) |
| >3 hospitalization | 1.00 (1.00–1.00) |
| 7-13 years after birth |  |
| 0 hospitalization | 1.00 (1.00–1.00) |
| 1-3 hospitalization | 1.00 (1.00–1.00) |
| >3 hospitalization | 1.00 (1.00–1.00) |
| 14-18 years after birth |  |
| 0 hospitalization | 1.00 (1.00–1.00) |
| 1-3 hospitalization | 1.00 (1.00–1.00) |
| >3 hospitalization | 1.00 (1.00–1.00) |
| Outpatient Clinic Utilization |  |
| 0-6 years after birth |  |
| 0 hospitalization | 1.02 (1.01–1.03) |
| 1-3 hospitalization | 0.99 (0.98–1.00) |
| >3 hospitalization | 0.96 (0.95–0.98) |
| 7-13 years after birth |  |
| 0 hospitalization | 1.01 (0.99–1.02) |
| 1-3 hospitalization | 0.97 (0.96–0.98) |
| >3 hospitalization | 0.96 (0.93–0.99) |
| 14-18 years after birth |  |
| 0 hospitalization | 1.03 (1.01–1.06) |
| 1-3 hospitalization | 0.98 (0.96–1.00) |
| >3 hospitalization | 0.93 (0.89–0.98) |
| Inpatient Care Utilization |  |
| 0-6 years after birth |  |
| 0 hospitalization | 1.10 (1.08–1.12) |
| 1-3 hospitalization | 1.05 (1.04–1.06) |
| >3 hospitalization | 1.02 (0.99–1.05) |
| 7-13 years after birth |  |
| 0 hospitalization | 1.05 (1.01–1.10) |
| 1-3 hospitalization | 0.96 (0.93–0.99) |
| >3 hospitalization | 0.89 (0.83–0.95) |
| 14-18 years after birth |  |
| 0 hospitalization | 1.12 (1.03–1.22) |
| 1-3 hospitalization | 0.90 (0.84–0.96) |
| >3 hospitalization | 0.93 (0.80–1.08) |
| Surgical Care Utilization |  |
| 0-6 years after birth |  |
| 0 hospitalization | 1.09 (1.06–1.11) |
| 1-3 hospitalization | 1.00 (0.98–1.02) |
| >3 hospitalization | 0.96 (0.92–1.00) |
| 7-13 years after birth |  |
| 0 hospitalization | 1.04 (1.00–1.07) |
| 1-3 hospitalization | 0.96 (0.94–0.99) |
| >3 hospitalization | 0.94 (0.87–1.00) |
| 14-18 years after birth |  |
| 0 hospitalization | 1.10 (1.03–1.18) |
| 1-3 hospitalization | 0.94 (0.89–0.99) |
| >3 hospitalization | inestimable |
| Psychiatric Clinic Utilization |  |
| 0-6 years after birth |  |
| 0 hospitalization | 1.28 (1.13–1.44) |
| 1-3 hospitalization | 0.81 (0.74–0.89) |
| >3 hospitalization | inestimable |
| 7-13 years after birth |  |
| 0 hospitalization | 1.18 (1.02–1.37) |
| 1-3 hospitalization | 0.88 (0.79–0.99) |
| >3 hospitalization | 0.78 (0.61–1.01) |
| 14-18 years after birth |  |
| 0 hospitalization | 1.24 (0.97–1.59) |
| 1-3 hospitalization | 0.87 (0.72–1.06) |
| >3 hospitalization | inestimable |
| Psychiatric Inpatient Care Utilization |  |
| 0-6 years after birth |  |
| 0 hospitalization | 1.23 (0.97–1.57) |
| 1-3 hospitalization | 0.74 (0.62–0.89) |
| >3 hospitalization | Inestimable |
| 7-13 years after birth |  |
| 0 hospitalization | 1.50 (1.17–1.93) |
| 1-3 hospitalization | 0.77 (0.62–0.95) |
| >3 hospitalization | Inestimable |
| 14-18 years after birth |  |
| 0 hospitalization | Inestimable |
| 1-3 hospitalization | Inestimable |
| >3 hospitalization | Inestimable |

**eAppendix 8.** Healthcare Utilization by the Number of Child Hospitalizations within the First Year: Quantity of Services Among Mothers Who Gave Birth to an Infant with a Major Congenital Anomalies (Exposed Cohort) and a Matched Comparison Cohort

|  | Adjusted Rate Ratio (95% CI) |
| --- | --- |
| Primary Care Utilization |  |
| 0-6 years after birth |  |
| 0 hospitalization | 1.05 (1.03–1.07) |
| 1-3 hospitalization | 0.94 (0.92–0.95) |
| >3 hospitalization | 0.82 (0.79–0.86) |
| 7-13 years after birth |  |
| 0 hospitalization | 1.05 (1.02–1.07) |
| 1-3 hospitalization | 0.94 (0.92–0.96) |
| >3 hospitalization | 0.86 (0.81–0.91) |
| 14-18 years after birth |  |
| 0 hospitalization | 1.03 (0.98–1.08) |
| 1-3 hospitalization | 0.96 (0.93–1.00) |
| >3 hospitalization | 0.94 (0.84–1.04) |
| Outpatient Clinic Utilization |  |
| 0-6 years after birth |  |
| 0 hospitalization | 1.30 (1.26–1.34) |
| 1-3 hospitalization | 0.99 (0.97–1.02) |
| >3 hospitalization | 0.85 (0.80–0.90) |
| 7-13 years after birth |  |
| 0 hospitalization | 1.12 (1.06–1.18) |
| 1-3 hospitalization | 0.95 (0.91–0.99) |
| >3 hospitalization | 0.85 (0.75–0.95) |
| 14-18 years after birth |  |
| 0 hospitalization | 1.11 (1.01–1.21) |
| 1-3 hospitalization | 0.92 (0.86–0.99) |
| >3 hospitalization | 0.95 (0.80–1.13) |
| Inpatient Care Utilization |  |
| 0-6 years after birth |  |
| 0 hospitalization | 1.24 (1.20–1.28) |
| 1-3 hospitalization | 1.11 (1.08–1.14) |
| >3 hospitalization | 0.95 (0.88–1.02) |
| 7-13 years after birth |  |
| 0 hospitalization | 1.08 (1.01–1.15) |
| 1-3 hospitalization | 0.93 (0.88–0.98) |
| >3 hospitalization | 0.75 (0.65–0.87) |
| 14-18 years after birth |  |
| 0 hospitalization | 1.11 (0.96–1.29) |
| 1-3 hospitalization | 0.87 (0.78–0.97) |
| >3 hospitalization | 0.87 (0.68–1.12) |
| Surgical Care Utilization |  |
| 0-6 years after birth |  |
| 0 hospitalization | 1.20 (1.15–1.24) |
| 1-3 hospitalization | 1.01 (0.98–1.04) |
| >3 hospitalization | 0.82 (0.75–0.89) |
| 7-13 years after birth |  |
| 0 hospitalization | 1.12 (1.05–1.19) |
| 1-3 hospitalization | 0.98 (0.94–1.03) |
| >3 hospitalization | 0.88 (0.78–1.00) |
| 14-18 years after birth |  |
| 0 hospitalization | 1.15 (1.03–1.29) |
| 1-3 hospitalization | 0.91 (0.83–0.99) |
| >3 hospitalization | Inestimable |
| Psychiatric Clinic Utilization |  |
| 0-6 years after birth |  |
| 0 hospitalization | 1.17 (0.93–1.48) |
| 1-3 hospitalization | 0.73 (0.58–0.92) |
| >3 hospitalization | inestimable |
| 7-13 years after birth |  |
| 0 hospitalization | 1.44 (1.07–1.95) |
| 1-3 hospitalization | 1.07 (0.81–1.41) |
| >3 hospitalization | 0.79 (0.47–1.32) |
| 14-18 years after birth |  |
| 0 hospitalization | 1.56 (0.87–2.79) |
| 1-3 hospitalization | 0.88 (0.57–1.36) |
| >3 hospitalization | inestimable |
| Psychiatric Inpatient Care Utilization |  |
| 0-6 years after birth |  |
| 0 hospitalization | 1.20 (0.83–1.74) |
| 1-3 hospitalization | 0.78 (0.57–1.05) |
| >3 hospitalization | inestimable |
| 7-13 years after birth |  |
| 0 hospitalization | 1.65 (1.09–2.51) |
| 1-3 hospitalization | 0.78 (0.51–1.21) |
| >3 hospitalization | inestimable |
| 14-18 years after birth |  |
| 0 hospitalization | inestimable |
| 1-3 hospitalization | inestimable |
| >3 hospitalization | inestimable |

**eAppendix 9.** Healthcare Utilization by Income: Period Prevalence Among Mothers Who Gave Birth to an Infant with a Major Congenital Anomalies (Exposed Cohort) and a Matched Comparison Cohort

|  | Adjusted Risk Ratio (95% CI) |
| --- | --- |
| Primary Care Utilization |  |
| 0-6 years after birth |  |
| Income Q1 | 1.00 (1.00–1.00) |
| Income Q2 | 1.00 (1.00–1.00) |
| Income Q3 | 1.00 (1.00–1.00) |
| Income Q4 | 1.00 (1.00–1.00) |
| 7-13 years after birth |  |
| Income Q1 | 1.00 (1.00–1.00) |
| Income Q2 | 1.00 (1.00–1.00) |
| Income Q3 | 1.00 (1.00–1.00) |
| Income Q4 | 1.00 (1.00–1.00) |
| 14-18 years after birth |  |
| Income Q1 | 1.00 (1.00–1.00) |
| Income Q2 | 1.00 (1.00–1.00) |
| Income Q3 | 1.00 (1.00–1.00) |
| Income Q4 | 1.00 (1.00–1.00) |
| Outpatient Clinic Utilization |  |
| 0-6 years after birth |  |
| Income Q1 | 1.02 (1.01–1.02) |
| Income Q2 | 1.02 (1.01–1.03) |
| Income Q3 | 1.01 (1.00–1.02) |
| Income Q4 | 1.02 (1.00–1.03) |
| 7-13 years after birth |  |
| Income Q1 | 1.01 (1.00–1.03) |
| Income Q2 | 1.00 (0.99–1.02) |
| Income Q3 | 1.02 (1.00–1.04) |
| Income Q4 | 1.01 (0.99–1.03) |
| 14-18 years after birth |  |
| Income Q1 | 1.05 (1.02–1.08) |
| Income Q2 | 1.01 (0.98–1.04) |
| Income Q3 | 1.04 (1.01–1.07) |
| Income Q4 | 1.01 (0.98–1.04) |
| Inpatient Care Utilization |  |
| 0-6 years after birth |  |
| Income Q1 | 1.09 (1.07–1.10) |
| Income Q2 | 1.14 (1.12–1.16) |
| Income Q3 | 1.15 (1.13–1.17) |
| Income Q4 | 1.13 (1.11–1.15) |
| 7-13 years after birth |  |
| Income Q1 | 1.07 (1.04–1.11) |
| Income Q2 | 1.05 (1.01–1.09) |
| Income Q3 | 1.07 (1.02–1.12) |
| Income Q4 | 1.12 (1.07–1.18) |
| 14-18 years after birth |  |
| Income Q1 | 1.14 (1.07–1.23) |
| Income Q2 | 1.06 (0.97–1.15) |
| Income Q3 | 1.11 (1.01–1.22) |
| Income Q4 | 1.02 (0.92–1.14) |
| Surgical Care Utilization |  |
| 0-6 years after birth |  |
| Income Q1 | 1.06 (1.03–1.08) |
| Income Q2 | 1.06 (1.04–1.08) |
| Income Q3 | 1.05 (1.02–1.07) |
| Income Q4 | 1.07 (1.04–1.09) |
| 7-13 years after birth |  |
| Income Q1 | 1.06 (1.02–1.09) |
| Income Q2 | 1.01 (0.98–1.05) |
| Income Q3 | 1.03 (0.99–1.07) |
| Income Q4 | 1.07 (1.02–1.12) |
| 14-18 years after birth |  |
| Income Q1 | 1.10 (1.03–1.17) |
| Income Q2 | 1.00 (0.93–1.07) |
| Income Q3 | 1.11 (1.04–1.19) |
| Income Q4 | 1.01 (0.93–1.09) |
| Psychiatric Clinic Utilization |  |
| 0-6 years after birth |  |
| Income Q1 | 1.16 (1.06–1.27) |
| Income Q2 | 1.10 (0.98–1.24) |
| Income Q3 | 1.14 (0.96–1.35) |
| Income Q4 | 1.11 (0.89–1.37) |
| 7-13 years after birth |  |
| Income Q1 | 1.15 (1.04–1.29) |
| Income Q2 | 1.04 (0.90–1.21) |
| Income Q3 | 1.19 (0.98–1.45) |
| Income Q4 | 1.01 (0.76–1.34) |
| 14-18 years after birth |  |
| Income Q1 | 1.25 (1.05–1.50) |
| Income Q2 | 0.96 (0.74–1.23) |
| Income Q3 | inestimable |
| Income Q4 | inestimable |
| Psychiatric Inpatient Care Utilization |  |
| 0-6 years after birth |  |
| Income Q1 | 1.23 (1.04–1.46) |
| Income Q2 | 0.78 (0.60–1.02) |
| Income Q3 | 0.87 (0.61–1.26) |
| Income Q4 | 1.29 (0.86–1.92) |
| 7-13 years after birth |  |
| Income Q1 | 1.23 (1.00–1.50) |
| Income Q2 | 1.17 (0.90–1.51) |
| Income Q3 | inestimable |
| Income Q4 | 1.07 (0.67–1.72) |
| 14-18 years after birth |  |
| Income Q1 | inestimable |
| Income Q2 | inestimable |
| Income Q3 | inestimable |
| Income Q4 | inestimable |

**eAppendix 10.** Healthcare Utilization by Income: Quantity of Services Among Mothers Who Gave Birth to an Infant with a Major Congenital Anomalies (Exposed Cohort) and a Matched Comparison Cohort

|  | Adjusted Rate Ratio (95% CI) |
| --- | --- |
| Primary Care Utilization |  |
| 0-6 years after birth |  |
| Income Q1 | 1.04 (1.02–1.07) |
| Income Q2 | 1.06 (1.04–1.08) |
| Income Q3 | 1.03 (1.02–1.05) |
| Income Q4 | 1.04 (1.02–1.06) |
| 7-13 years after birth |  |
| Income Q1 | 1.07 (1.04–1.10) |
| Income Q2 | 1.06 (1.03–1.09) |
| Income Q3 | 1.04 (1.01–1.07) |
| Income Q4 | 1.02 (0.99–1.05) |
| 14-18 years after birth |  |
| Income Q1 | 1.09 (1.04–1.15) |
| Income Q2 | 1.03 (0.99–1.09) |
| Income Q3 | 1.08 (1.03–1.14) |
| Income Q4 | 1.04 (0.99–1.09) |
| Outpatient Clinic Utilization |  |
| 0-6 years after birth |  |
| Income Q1 | 1.18 (1.14–1.21) |
| Income Q2 | 1.23 (1.19–1.27) |
| Income Q3 | 1.16 (1.12–1.20) |
| Income Q4 | 1.20 (1.16–1.25) |
| 7-13 years after birth |  |
| Income Q1 | 1.13 (1.08–1.19) |
| Income Q2 | 1.13 (1.07–1.20) |
| Income Q3 | 1.06 (1.00–1.12) |
| Income Q4 | 1.18 (1.10–1.27) |
| 14-18 years after birth |  |
| Income Q1 | 1.18 (1.09–1.28) |
| Income Q2 | 1.09 (1.00–1.18) |
| Income Q3 | 1.09 (0.99–1.20) |
| Income Q4 | 1.08 (0.97–1.20) |
| Inpatient Care Utilization |  |
| 0-6 years after birth |  |
| Income Q1 | 1.34 (1.29–1.38) |
| Income Q2 | 1.41 (1.36–1.47) |
| Income Q3 | 1.42 (1.37–1.48) |
| Income Q4 | 1.40 (1.35–1.45) |
| 7-13 years after birth |  |
| Income Q1 | 1.17 (1.10–1.25) |
| Income Q2 | 1.14 (1.06–1.21) |
| Income Q3 | 1.13 (1.04–1.22) |
| Income Q4 | 1.13 (1.05–1.23) |
| 14-18 years after birth |  |
| Income Q1 | 1.23 (1.08–1.40) |
| Income Q2 | 1.11 (0.97–1.27) |
| Income Q3 | 1.12 (0.94–1.32) |
| Income Q4 | 1.03 (0.87–1.23) |
| Surgical Care Utilization |  |
| 0-6 years after birth |  |
| Income Q1 | 1.16 (1.11–1.20) |
| Income Q2 | 1.18 (1.13–1.23) |
| Income Q3 | 1.11 (1.07–1.16) |
| Income Q4 | 1.15 (1.10–1.20) |
| 7-13 years after birth |  |
| Income Q1 | 1.14 (1.07–1.21) |
| Income Q2 | 1.09 (1.03–1.17) |
| Income Q3 | 1.07 (1.00–1.15) |
| Income Q4 | 1.11 (1.03–1.20) |
| 14-18 years after birth |  |
| Income Q1 | 1.15 (1.03–1.28) |
| Income Q2 | 1.10 (0.98–1.23) |
| Income Q3 | 1.09 (0.97–1.21) |
| Income Q4 | 1.01 (0.89–1.16) |
| Psychiatric Clinic Utilization |  |
| 0-6 years after birth |  |
| Income Q1 | 1.21 (0.99–1.47) |
| Income Q2 | 1.10 (0.89–1.37) |
| Income Q3 | 0.80 (0.60–1.08) |
| Income Q4 | 1.09 (0.68-1.73) |
| 7-13 years after birth |  |
| Income Q1 | 1.59 (1.24–2.03) |
| Income Q2 | 0.94 (0.72–1.23) |
| Income Q3 | 1.28 (0.89–1.84) |
| Income Q4 | 1.04 (0.63–1.74) |
| 14-18 years after birth |  |
| Income Q1 | 1.28 (0.90–1.83) |
| Income Q2 | 0.85 (0.56–1.29) |
| Income Q3 | Inestimable |
| Income Q4 | Inestimable |
| Psychiatric Inpatient Care Utilization |  |
| 0-6 years after birth |  |
| Income Q1 | 1.57 (1.15–2.15) |
| Income Q2 | 0.74 (0.52–1.05) |
| Income Q3 | 0.77 (0.49–1.20) |
| Income Q4 | 1.56 (0.93–2.63) |
| 7-13 years after birth |  |
| Income Q1 | 1.76 (1.07–2.89) |
| Income Q2 | 0.91 (0.60–1.36) |
| Income Q3 | Inestimable |
| Income Q4 | 0.70 (0.41–1.21) |
| 14-18 years after birth |  |
| Income Q1 | Inestimable |
| Income Q2 | inestimable |
| Income Q3 | inestimable |
| Income Q4 | inestimable |

**eAppendix 11.** Healthcare Utilization by MCA-related history: Period Prevalence Among Mothers Who Gave Birth to an Infant with a Major Congenital Anomalies (Exposed Cohort) and a Matched Comparison Cohort

|  | Adjusted Risk Ratio (95% CI) |
| --- | --- |
| Primary Care Utilization |  |
| 0-6 years after birth |  |
| Single MCA pregnancy history | 1.00 (1.00–1.00) |
| Multiple MCA pregnancy histories | 1.00 (1.00–1.00) |
| 7-13 years after birth |  |
| Single MCA pregnancy history | 1.00 (1.00–1.00) |
| Multiple MCA pregnancy histories | 1.00 (1.00–1.00) |
| 14-18 years after birth |  |
| Single MCA pregnancy history | 1.00 (1.00–1.00) |
| Multiple MCA pregnancy histories | 1.00 (1.00–1.00) |
| Outpatient Clinic Utilization |  |
| 0-6 years after birth |  |
| Single MCA pregnancy history | 1.01 (1.01–1.02) |
| Multiple MCA pregnancy histories | 1.07 (1.06–1.09) |
| 7-13 years after birth |  |
| Single MCA pregnancy history | 1.01 (1.00–1.02) |
| Multiple MCA pregnancy histories | 1.06 (1.02–1.11) |
| 14-18 years after birth |  |
| Single MCA pregnancy history | 1.02 (1.01–1.04) |
| Multiple MCA pregnancy histories | 1.09 (1.01–1.17) |
| Inpatient Care Utilization |  |
| 0-6 years after birth |  |
| Single MCA pregnancy history | 1.12 (1.11–1.13) |
| Multiple MCA pregnancy histories | 1.25 (1.22–1.29) |
| 7-13 years after birth |  |
| Single MCA pregnancy history | 1.07 (1.05–1.09) |
| Multiple MCA pregnancy histories | 1.23 (1.13–1.35) |
| 14-18 years after birth |  |
| Single MCA pregnancy history | 1.09 (1.04–1.14) |
| Multiple MCA pregnancy histories | 1.17 (0.95–1.44) |
| Surgical Care Utilization |  |
| 0-6 years after birth |  |
| Single MCA pregnancy history | 1.05 (1.04–1.07) |
| Multiple MCA pregnancy histories | 1.26 (1.20–1.32) |
| 7-13 years after birth |  |
| Single MCA pregnancy history | 1.04 (1.02–1.06) |
| Multiple MCA pregnancy histories | 1.19 (1.10–1.30) |
| 14-18 years after birth |  |
| Single MCA pregnancy history | 1.05 (1.01–1.09) |
| Multiple MCA pregnancy histories | 1.08 (0.90–1.30) |
| Psychiatric Clinic Utilization |  |
| 0-6 years after birth |  |
| Single MCA pregnancy history | 1.14 (1.07–1.22) |
| Multiple MCA pregnancy histories | inestimable |
| 7-13 years after birth |  |
| Single MCA pregnancy history | 1.11 (1.03–1.20) |
| Multiple MCA pregnancy histories | inestimable |
| 14-18 years after birth |  |
| Single MCA pregnancy history | 1.10 (0.96–1.26) |
| Multiple MCA pregnancy histories | inestimable |
| Psychiatric Inpatient Care Utilization |  |
| 0-6 years after birth |  |
| Single MCA pregnancy history | 1.05 (0.92–1.19) |
| Multiple MCA pregnancy histories | inestimable |
| 7-13 years after birth |  |
| Single MCA pregnancy history | 1.20 (1.05–1.38) |
| Multiple MCA pregnancy histories | inestimable |
| 14-18 years after birth |  |
| Single MCA pregnancy history | inestimable |
| Multiple MCA pregnancy histories | inestimable |

**eAppendix 12.** Healthcare Utilization by MCA-related history: Quantity of Services Among Mothers Who Gave Birth to an Infant with a Major Congenital Anomalies (Exposed Cohort) and a Matched Comparison Cohort

|  | Adjusted Rate Ratio (95% CI) |
| --- | --- |
| Primary Care Utilization |  |
| 0-6 years after birth |  |
| Single MCA pregnancy history | 1.04 (1.03–1.05) |
| Multiple MCA pregnancy histories | 1.14 (1.08–1.21) |
| 7-13 years after birth |  |
| Single MCA pregnancy history | 1.05 (1.03–1.06) |
| Multiple MCA pregnancy histories | 1.09 (1.02–1.18) |
| 14-18 years after birth |  |
| Single MCA pregnancy history | 1.07 (1.04–1.09) |
| Multiple MCA pregnancy histories | 0.99 (0.88–1.11) |
| Outpatient Clinic Utilization |  |
| 0-6 years after birth |  |
| Single MCA pregnancy history | 1.18 (1.16–1.20) |
| Multiple MCA pregnancy histories | 1.83 (1.69–1.97) |
| 7-13 years after birth |  |
| Single MCA pregnancy history | 1.11 (1.08–1.15) |
| Multiple MCA pregnancy histories | 1.48 (1.28–1.71) |
| 14-18 years after birth |  |
| Single MCA pregnancy history | 1.11 (1.06–1.16) |
| Multiple MCA pregnancy histories | 1.09 (0.89–1.33) |
| Inpatient Care Utilization |  |
| 0-6 years after birth |  |
| Single MCA pregnancy history | 1.38 (1.35–1.40) |
| Multiple MCA pregnancy histories | 1.85 (1.71–2.01) |
| 7-13 years after birth |  |
| Single MCA pregnancy history | 1.13 (1.09–1.17) |
| Multiple MCA pregnancy histories | 1.54 (1.30–1.81) |
| 14-18 years after birth |  |
| Single MCA pregnancy history | 1.12 (1.04–1.21) |
| Multiple MCA pregnancy histories | 1.33 (0.94–1.89) |
| Surgical Care Utilization |  |
| 0-6 years after birth |  |
| Single MCA pregnancy history | 1.14 (1.11–1.16) |
| Multiple MCA pregnancy histories | 1.61 (1.47–1.77) |
| 7-13 years after birth |  |
| Single MCA pregnancy history | 1.09 (1.06–1.13) |
| Multiple MCA pregnancy histories | 1.50 (1.28–1.75) |
| 14-18 years after birth |  |
| Single MCA pregnancy history | 1.08 (1.02–1.15) |
| Multiple MCA pregnancy histories | 1.28 (0.95–1.71) |
| Psychiatric Clinic Utilization |  |
| 0-6 years after birth |  |
| Single MCA pregnancy history | 1.05 (0.91–1.22) |
| Multiple MCA pregnancy histories | Inestimable |
| 7-13 years after birth |  |
| Single MCA pregnancy history | 1.23 (1.03–1.47) |
| Multiple MCA pregnancy histories | Inestimable |
| 14-18 years after birth |  |
| Single MCA pregnancy history | 1.16 (0.86–1.56) |
| Multiple MCA pregnancy histories | Inestimable |
| Psychiatric Inpatient Care Utilization |  |
| 0-6 years after birth |  |
| Single MCA pregnancy history | 1.12 (0.91–1.38) |
| Multiple MCA pregnancy histories | Inestimable |
| 7-13 years after birth |  |
| Single MCA pregnancy history | 1.23 (0.93–1.61) |
| Multiple MCA pregnancy histories | inestimable |
| 14-18 years after birth |  |
| Single MCA pregnancy history | inestimable |
| Multiple MCA pregnancy histories | inestimable |

**eAppendix 13.** Healthcare Utilization by the Modified Charlson Comorbidity Index (CCI) Score: Period Prevalence Among Mothers Who Gave Birth to an Infant with a Major Congenital Anomalies (Exposed Cohort) and a Matched Comparison Cohort

|  | Adjusted Risk Ratio (95% CI) |
| --- | --- |
| Primary Care Utilization |  |
| 0-6 years after birth |  |
| CCI Score 0 | 1.00 (1.00–1.00) |
| CCI Score ≥ 1 | 1.00 (1.00–1.00) |
| 7-13 years after birth |  |
| CCI Score 0 | 1.00 (1.00–1.00) |
| CCI Score ≥ 1 | 1.00 (1.00–1.00) |
| 14-18 years after birth |  |
| CCI Score 0 | 1.00 (1.00–1.00) |
| CCI Score ≥ 1 | 0.99 (0.98–1.01) |
| Outpatient Clinic Utilization |  |
| 0-6 years after birth |  |
| CCI Score 0 | 1.02 (1.02–1.03) |
| CCI Score ≥ 1 | 1.02 (1.01–1.03) |
| 7-13 years after birth |  |
| CCI Score 0 | 1.01 (1.01–1.02) |
| CCI Score ≥ 1 | 1.02 (0.99–1.05) |
| 14-18 years after birth |  |
| CCI Score 0 | 1.03 (1.01–1.04) |
| CCI Score ≥ 1 | 1.05 (1.00–1.09) |
| Inpatient Care Utilization |  |
| 0-6 years after birth |  |
| CCI Score 0 | 1.14 (1.13–1.15) |
| CCI Score ≥ 1 | 1.09 (1.05–1.12) |
| 7-13 years after birth |  |
| CCI Score 0 | 1.08 (1.05–1.10) |
| CCI Score ≥ 1 | 1.09 (1.01–1.17) |
| 14-18 years after birth |  |
| CCI Score 0 | 1.07 (1.03–1.12) |
| CCI Score ≥ 1 | 1.15 (0.99–1.35) |
| Surgical Care Utilization |  |
| 0-6 years after birth |  |
| CCI Score 0 | 1.07 (1.06–1.08) |
| CCI Score ≥ 1 | 1.05 (1.01–1.09) |
| 7-13 years after birth |  |
| CCI Score 0 | 1.04 (1.02–1.06) |
| CCI Score ≥ 1 | 1.14 (1.06–1.22) |
| 14-18 years after birth |  |
| CCI Score 0 | 1.06 (1.02–1.10) |
| CCI Score ≥ 1 | 1.04 (0.91–1.20) |
| Psychiatric Clinic Utilization |  |
| 0-6 years after birth |  |
| CCI Score 0 | 1.16 (1.09–1.24) |
| CCI Score ≥ 1 | inestimable |
| 7-13 years after birth |  |
| CCI Score 0 | 1.18 (1.09–1.27) |
| CCI Score ≥ 1 | inestimable |
| 14-18 years after birth |  |
| CCI Score 0 | 1.11 (0.98–1.27) |
| CCI Score ≥ 1 | inestimable |
| Psychiatric Inpatient Care Utilization |  |
| 0-6 years after birth |  |
| CCI Score 0 | 1.09 (0.96–1.24) |
| CCI Score ≥ 1 | inestimable |
| 7-13 years after birth |  |
| CCI Score 0 | 1.24 (1.07–1.42) |
| CCI Score ≥ 1 | 1.06 (0.62–1.80) |
| 14-18 years after birth |  |
| CCI Score 0 | 1.02 (0.80–1.31) |
| CCI Score ≥ 1 | inestimable |

**eAppendix 14.** Healthcare Utilization by the Modified Charlson Comorbidity Index (CCI) Score: Quantity of Services Among Mothers Who Gave Birth to an Infant with a Major Congenital Anomalies (Exposed Cohort) and a Matched Comparison Cohort

|  | Adjusted Rate Ratio (95% CI) |
| --- | --- |
| Primary Care Utilization |  |
| 0-6 years after birth |  |
| CCI Score 0 | 1.05 (1.04–1.06) |
| CCI Score ≥ 1 | 1.03 (0.99–1.07) |
| 7-13 years after birth |  |
| CCI Score 0 | 1.05 (1.04–1.06) |
| CCI Score ≥ 1 | 1.03 (0.97–1.10) |
| 14-18 years after birth |  |
| CCI Score 0 | 1.06 (1.04–1.09) |
| CCI Score ≥ 1 | 1.08 (0.96–1.22) |
| Outpatient Clinic Utilization |  |
| 0-6 years after birth |  |
| CCI Score 0 | 1.22 (1.20–1.24) |
| CCI Score ≥ 1 | 1.18 (1.10–1.26) |
| 7-13 years after birth |  |
| CCI Score 0 | 1.15 (1.12–1.18) |
| CCI Score ≥ 1 | 1.17 (1.03–1.34) |
| 14-18 years after birth |  |
| CCI Score 0 | 1.11 (1.06–1.16) |
| CCI Score ≥ 1 | 1.14 (0.94–1.38) |
| Inpatient Care Utilization |  |
| 0-6 years after birth |  |
| CCI Score 0 | 1.41 (1.39–1.44) |
| CCI Score ≥ 1 | 1.31 (1.21–1.42) |
| 7-13 years after birth |  |
| CCI Score 0 | 1.15 (1.11–1.20) |
| CCI Score ≥ 1 | 1.16 (1.01–1.35) |
| 14-18 years after birth |  |
| CCI Score 0 | 1.11 (1.04–1.20) |
| CCI Score ≥ 1 | 1.13 (0.81–1.58) |
| Surgical Care Utilization |  |
| 0-6 years after birth |  |
| CCI Score 0 | 1.17 (1.14–1.19) |
| CCI Score ≥ 1 | 1.12 (1.03–1.21) |
| 7-13 years after birth |  |
| CCI Score 0 | 1.13 (1.09–1.16) |
| CCI Score ≥ 1 | 1.07 (0.94–1.22) |
| 14-18 years after birth |  |
| CCI Score 0 | 1.11 (1.05–1.18) |
| CCI Score ≥ 1 | 0.94 (0.73–1.21) |
| Psychiatric Clinic Utilization |  |
| 0-6 years after birth |  |
| CCI Score 0 | 1.11 (0.95–1.29) |
| CCI Score ≥ 1 | inestimable |
| 7-13 years after birth |  |
| CCI Score 0 | 1.28 (1.07–1.53) |
| CCI Score ≥ 1 | inestimable |
| 14-18 years after birth |  |
| CCI Score 0 | 1.04 (0.78–1.39) |
| CCI Score ≥ 1 | inestimable |
| Psychiatric Inpatient Care Utilization |  |
| 0-6 years after birth |  |
| CCI Score 0 | 1.14 (0.93–1.41) |
| CCI Score ≥ 1 | inestimable |
| 7-13 years after birth |  |
| CCI Score 0 | 1.27 (0.97–1.66) |
| CCI Score ≥ 1 | 1.32 (0.62–2.81) |
| 14-18 years after birth |  |
| CCI Score 0 | 1.40 (0.77–2.54) |
| CCI Score ≥ 1 | inestimable |
