# Supplementary material for: Mothers of children with major congenital anomalies have increased health care utilization over a 20-year post-birth time horizon
Source: PLoS One. 2021 Dec 8;16(12):e0260962. doi: 10.1371/journal.pone.0260962 (PMC8654179; doi:10.1371/journal.pone.0260962)
Supplement: Attachment — Submitted filename: Response to Reviewers.docx [file pone.0260962.s002.docx]

October 15, 2021

Angela Lupattelli, PhD

Academic Editor

Dear Dr. Lupatteli,

We thank the Editor and Reviewers for their thoughtful comments and appreciate the opportunity to revise our manuscript. We believe the manuscript has substantially benefited as a result.

The major changes in this revision are summarized below:

1. We expanded substantially upon the sensitivity analyses to address concerns about (1) the definition of major congenital anomalies used, which covers a wide variety of prognoses, and (2) the fact that increased health care utilization might have been related to the mother’s own health conditions.
2. We revised the manuscript’s text to emphasize that the limitations of the study include potential sources of bias from a high proportion of mothers without cohabitants, and from many statistical comparisons.
3. We evaluated the methods section carefully. To clarify our cohort selection procedure, we updated the study cohort information about the percentage of mothers who were matched multiple times, and we updated Figure 1.

Our point-by-point responses to the comments are below. Reviewer comments are presented in italic text, and our responses and all changes in the revised text are presented in regular text.

**Reviewer #1 comments**

***Comment: I wonder about the high number of ‘singles/divorced/widowed/living alone’. It adds up to 44 and 47 % in the groups with and without CMA, respectively. Is it the case that cohabitants are left out? This is an important point as having a partner is of great importance when it comes to how people live through life's various challenges.***

Response: We agree that cohabitation plays an important role when analyzing health-related outcomes in mothers of children with MCAs. We cannot rule out the misclassification of marital status, since some women might not register their cohabitation status in the Danish Civil Registration System. In such a case, the women are included in the “singles/divorced/widowed/living alone” group. We added this limitation in the *Limitation* section. In addition, misclassification of marital status is not a big problem in Denmark because the Danish Civil Registration System uses tax forms.

Page 20.

We cannot rule out the misclassification of marital status because some mothers might not have registered their cohabitation status. This might lead to a high proportion of mothers with non-married status, and we could not examine the role of cohabitation in our study. However, if rates of misclassification are similar between the exposed and unexposed cohorts, this would not bias the results.

***Comment: Major congenital malformations cover a wide variety of prognoses. Some of these are "relatively straightforward", i.e., surgical corrected without sequelae (e. g. Q793, gastroschisis and Q792 omphalocele (unless combined with chromosome abnormalities)). It would be interesting to study this in more detail by removing this type of MCA from the analyzes to see if the results were changed.***

Response: We agree with the Reviewer that our definition of major congenital anomalies covers a wide range of diagnoses; however, we think that the diagnosis itself may not account for varied prognoses among individual diagnoses (e.g. for gastroschisis/omphalocele, whether or not the patient develops short bowel syndrome). Even a systematic review of all the analyzed MCA diagnoses codes would require specific judgments from clinicians and would have many limitations. To account for this potential problem, we performed a sensitivity analysis excluding all mothers with infants, who were not re-hospitalized after the newborn period in the first year of life as a proxy measure for how sick a baby was. Results from the analysis excluding less-sick children show that the mother’s health care utilization did not increase with increased severity of MCA, except for the inpatient admissions during the first 6 years after birth (adjusted risk ratio 1.06; 95% CI, 1.04-1.07, adjusted rate ratio 1.17; 95% CI, 1.14-1.19). This indicates that the mother’s health care utilization might not necessarily be associated with MCA severity, but with the burden of caring for children with MCA in general.

**Reviewer #3 Comments**

***Comment: 1) It is not clear whether mothers with children with MCA had increased rates of health care utilization prior to birth (or prior to pregnancy). It is possible that these women had other health problems that led to both higher risk of congenital anomaly in the offspring and increased use of health services. In fact, Figure 3 suggests elevated rates of health care visits were observed in these women 2 years prior to birth. This would affect the interpretation of your results.***

Response: We agree that mothers of children with MCA might have increased rates of health care utilization prior to birth.

1. We addressed this potential issue by adjusting for factors that are associated independently with elevated risk of increased use of health-care utilization in the original manuscript. These factors include chronic hypertension, diabetes, and alcohol use prior to the index pregnancy, and the modified Charlson comorbidity index score that excluded hypertension, diabetes, and alcohol use. We added the following text to state clearly that these covariates are adjusted.

Page 7.

Other maternal medical history was summarized using a modified Charlson comorbidity index score that excluded hypertension, diabetes, and alcohol use.

2. We agree that Figure 3 suggested a slightly elevated rate of health-care visits prior to birth among mothers of children with MCA. However, Figure 3 presents the annual mean health care service utilization before controlling for the covariates stated above. We modified the text to make it clearer.

Page 13.

Unadjusted annual mean health care service utilization is presented in Figure 3.

3. To address the concern further, we conducted the stratified analyses, comparing the mothers of children with MCA with the comparison cohort according to the health conditions that are independently associated with increased health-care utilization (i.e., hypertension, diabetes, alcohol use, and the modified Charlson comorbidity index score). We found an elevated risk of health care utilization among mothers of children with MCA and similar levels of medical conditions. We added the following text.

Page 8.

We also conducted stratified analyses to investigate whether other important risk factors, such as mothers’ health-related behaviors (*i.e*., alcohol use, smoking history) and mother’s own health conditions, were associated with increased health care utilization.

Page 15.

Increased health care utilization associated with mothers of children with MCA were observed for mothers with similar levels of medical conditions. Among mothers with the existing medical conditions (*i.e.,* the Modified Charlson Comorbidity Index ≥ 1), the exposed cohort used more outpatient clinic services, inpatients services, and surgical care services compared to the unexposed cohort (eAppendix 13-14).

4. We also added the tables presenting the results from the stratified analysis by the modified Charlson comorbidity index score in the Supplement. Other stratified analyses results are also available upon request.

**eAppendix 13.** Healthcare Utilization by the Modified Charlson Comorbidity Index (CCI) Score: Period Prevalence Among Mothers Who Gave Birth to an Infant with a Major Congenital Anomalies (Exposed Cohort) and a Matched Comparison Cohort

|  | Adjusted Risk Ratio (95% CI) |
| --- | --- |
| Primary Care Utilization |  |
| 0-6 years after birth |  |
| CCI Score 0 | 1.00 (1.00–1.00) |
| CCI Score ≥ 1 | 1.00 (1.00–1.00) |
| 7-13 years after birth |  |
| CCI Score 0 | 1.00 (1.00–1.00) |
| CCI Score ≥ 1 | 1.00 (1.00–1.00) |
| 14-18 years after birth |  |
| CCI Score 0 | 1.00 (1.00–1.00) |
| CCI Score ≥ 1 | 0.99 (0.98–1.01) |
| Outpatient Clinic Utilization |  |
| 0-6 years after birth |  |
| CCI Score 0 | 1.02 (1.02–1.03) |
| CCI Score ≥ 1 | 1.02 (1.01–1.03) |
| 7-13 years after birth |  |
| CCI Score 0 | 1.01 (1.01–1.02) |
| CCI Score ≥ 1 | 1.02 (0.99–1.05) |
| 14-18 years after birth |  |
| CCI Score 0 | 1.03 (1.01–1.04) |
| CCI Score ≥ 1 | 1.05 (1.00–1.09) |
| Inpatient Care Utilization |  |
| 0-6 years after birth |  |
| CCI Score 0 | 1.14 (1.13–1.15) |
| CCI Score ≥ 1 | 1.09 (1.05–1.12) |
| 7-13 years after birth |  |
| CCI Score 0 | 1.08 (1.05–1.10) |
| CCI Score ≥ 1 | 1.09 (1.01–1.17) |
| 14-18 years after birth |  |
| CCI Score 0 | 1.07 (1.03–1.12) |
| CCI Score ≥ 1 | 1.15 (0.99–1.35) |
| Surgical Care Utilization |  |
| 0-6 years after birth |  |
| CCI Score 0 | 1.07 (1.06–1.08) |
| CCI Score ≥ 1 | 1.05 (1.01–1.09) |
| 7-13 years after birth |  |
| CCI Score 0 | 1.04 (1.02–1.06) |
| CCI Score ≥ 1 | 1.14 (1.06–1.22) |
| 14-18 years after birth |  |
| CCI Score 0 | 1.06 (1.02–1.10) |
| CCI Score ≥ 1 | 1.04 (0.91–1.20) |
| Psychiatric Clinic Utilization |  |
| 0-6 years after birth |  |
| CCI Score 0 | 1.16 (1.09–1.24) |
| CCI Score ≥ 1 | inestimable |
| 7-13 years after birth |  |
| CCI Score 0 | 1.18 (1.09–1.27) |
| CCI Score ≥ 1 | inestimable |
| 14-18 years after birth |  |
| CCI Score 0 | 1.11 (0.98–1.27) |
| CCI Score ≥ 1 | inestimable |
| Psychiatric Inpatient Care Utilization |  |
| 0-6 years after birth |  |
| CCI Score 0 | 1.09 (0.96–1.24) |
| CCI Score ≥ 1 | inestimable |
| 7-13 years after birth |  |
| CCI Score 0 | 1.24 (1.07–1.42) |
| CCI Score ≥ 1 | 1.06 (0.62–1.80) |
| 14-18 years after birth |  |
| CCI Score 0 | 1.02 (0.80–1.31) |
| CCI Score ≥ 1 | inestimable |

**eAppendix 14.** Healthcare Utilization by the Modified Charlson Comorbidity Index (CCI) Score: Quantity of Services Among Mothers Who Gave Birth to an Infant with a Major Congenital Anomalies (Exposed Cohort) and a Matched Comparison Cohort

|  | Adjusted Rate Ratio (95% CI) |
| --- | --- |
| Primary Care Utilization |  |
| 0-6 years after birth |  |
| CCI Score 0 | 1.05 (1.04–1.06) |
| CCI Score ≥ 1 | 1.03 (0.99–1.07) |
| 7-13 years after birth |  |
| CCI Score 0 | 1.05 (1.04–1.06) |
| CCI Score ≥ 1 | 1.03 (0.97–1.10) |
| 14-18 years after birth |  |
| CCI Score 0 | 1.06 (1.04–1.09) |
| CCI Score ≥ 1 | 1.08 (0.96–1.22) |
| Outpatient Clinic Utilization |  |
| 0-6 years after birth |  |
| CCI Score 0 | 1.22 (1.20–1.24) |
| CCI Score ≥ 1 | 1.18 (1.10–1.26) |
| 7-13 years after birth |  |
| CCI Score 0 | 1.15 (1.12–1.18) |
| CCI Score ≥ 1 | 1.17 (1.03–1.34) |
| 14-18 years after birth |  |
| CCI Score 0 | 1.11 (1.06–1.16) |
| CCI Score ≥ 1 | 1.14 (0.94–1.38) |
| Inpatient Care Utilization |  |
| 0-6 years after birth |  |
| CCI Score 0 | 1.41 (1.39–1.44) |
| CCI Score ≥ 1 | 1.31 (1.21–1.42) |
| 7-13 years after birth |  |
| CCI Score 0 | 1.15 (1.11–1.20) |
| CCI Score ≥ 1 | 1.16 (1.01–1.35) |
| 14-18 years after birth |  |
| CCI Score 0 | 1.11 (1.04–1.20) |
| CCI Score ≥ 1 | 1.13 (0.81–1.58) |
| Surgical Care Utilization |  |
| 0-6 years after birth |  |
| CCI Score 0 | 1.17 (1.14–1.19) |
| CCI Score ≥ 1 | 1.12 (1.03–1.21) |
| 7-13 years after birth |  |
| CCI Score 0 | 1.13 (1.09–1.16) |
| CCI Score ≥ 1 | 1.07 (0.94–1.22) |
| 14-18 years after birth |  |
| CCI Score 0 | 1.11 (1.05–1.18) |
| CCI Score ≥ 1 | 0.94 (0.73–1.21) |
| Psychiatric Clinic Utilization |  |
| 0-6 years after birth |  |
| CCI Score 0 | 1.11 (0.95–1.29) |
| CCI Score ≥ 1 | inestimable |
| 7-13 years after birth |  |
| CCI Score 0 | 1.28 (1.07–1.53) |
| CCI Score ≥ 1 | inestimable |
| 14-18 years after birth |  |
| CCI Score 0 | 1.04 (0.78–1.39) |
| CCI Score ≥ 1 | inestimable |
| Psychiatric Inpatient Care Utilization |  |
| 0-6 years after birth |  |
| CCI Score 0 | 1.14 (0.93–1.41) |
| CCI Score ≥ 1 | inestimable |
| 7-13 years after birth |  |
| CCI Score 0 | 1.27 (0.97–1.66) |
| CCI Score ≥ 1 | 1.32 (0.62–2.81) |
| 14-18 years after birth |  |
| CCI Score 0 | 1.40 (0.77–2.54) |
| CCI Score ≥ 1 | inestimable |

5. We added this as a potential source of bias in the Limitation section.

Page 19.

The observed associations between mothers of children with MCA and health care utilization might be slightly overestimated because the exposed cohort might present with worse health conditions prior to the index birth. To address this issue, we adjusted for factors independently associated with increased use of health care services. We also performed the stratified analyses by these factors and confirmed the robustness of our main results.

***Comment: Page 5: How many women were matched multiple times? (if matching was with replacement)***

Response: Thank you for this important question. Matching was done with replacement and 15.5% of all women from the comparison cohort were used more than once. We added this information to the *Methods* section. To account for repeated measures in the same individuals, in the modified Poisson and negative binomial regression models we computed the 95% confidence intervals using a robust standard error estimation. We added the following to the Study cohorts section.

Page 5.

Matching was done with replacement[13] and 15.5% of all women from the comparison cohort were used more than once.

***Comment: Page 6: “…non-placental complications (including intrauterine hypoxia/birth asphyxia,…” Intrauterine hypoxia and fetal-maternal hemorrhage can be also caused by placental complications (e.g., placental insufficiency, abruption). The dichotomy between ‘placental’ and ‘non-placental’ complications is ambiguous.***

Response: We adjusted complications during the index pregnancy, including placental and non-placental complications according to our prior published studies (Cohen et al. 2016, 2018). Although we agree that the dichotomy between the two may be ambiguous, the bias related to the ambiguity is minimal when we adjust for all these complications.

- Cohen, Eyal, Erzsébet Horváth-Puhó, Joel G. Ray, Lars Pedersen, Nancy Adler, Anne Gulbech Ording, Paul H. Wise, Arnold Milstein, and Henrik Toft Sørensen. 2016. “Association between the Birth of an Infant with Major Congenital Anomalies and Subsequent Risk of Mortality in Their Mothers.” *JAMA: The Journal of the American Medical Association* 316 (23): 2515.
- Cohen, Eyal, Erzsébet Horváth-Puhó, Joel G. Ray, Lars Pedersen, Vera Ehrenstein, Nancy Adler, Simone Vigod, Arnold Milstein, and Henrik Toft Sørensen. 2018. “Cardiovascular Disease among Women Who Gave Birth to an Infant with a Major Congenital Anomaly.” *JAMA Network Open* 1 (5): e182320.

***Comment: Another limitation to be discussed in the Discussion: Many statistical comparisons can lead to false positive results.***

Response: We understand and appreciate the Reviewer’s request, but we, respectfully, prefer to refrain from focusing on specific statistical comparisons in the manuscript. P-values and statistical significance testing have been misused for decades in academic literature and recent literature continues to emphasize this point (Amrhein V, et al. Scientists rise up against statistical significance. Nature. 2019;567:305–307). We prefer to focus on the actual effect size and confidence intervals instead of focusing on statistical significance and multiple comparisons. Our choice is supported by recommendations in the Strengthening the Reporting of Observational Studies in Epidemiology (STROBE) guidelines (Von Elm E, Altman DG, Egger M, et al. The Strengthening the Reporting of Observational Studies in Epidemiology (STROBE) statement: guidelines for reporting observational studies. Bull World Health Organ. 2007;85:867–872.).

***Comment: Figure 3. Labels of Y-axes are not entirely clear. Did you mean, for instance, ‘Average number of visits per person per year’, or ‘Average number of hospitalizations per person per year’?***

Response: We changed the labels of the Y-axes as follows.

Figure 3.

Annual Mean of Primary Care Visits per Person

Annual Mean of Outpatient Clinic Visits per Person

Annual Mean of Inpatient Admissions per Person

Annual Mean of Surgeries per Person

Annual Mean of Outpatient Psychiatric Clinic Visits per Person

Annual Mean of Psychiatric Inpatient Admissions per Person

***Comment: Some scales on y-axes (0-2) can be more granular.***

Response: We changed the scale on the Y-axes, ranging from 0 to 2, increased by 0.5 numbers, for inpatient admissions and surgical care service utilization in Figure 3.

Figure 3.

***Comment: Figure 1: How many matched women in the comparison group had missing values or were excluded?***

Response: Comparison cohort members were randomly selected from a cohort of eligible women (n = 1,273,917), i.e. after exclusion of mothers with missing data on key variables and matching factors (total 63,016 pregnancies were excluded). Therefore, the matched comparison group (n = 239,076) had no missing data and further exclusion was not needed after matching.

***Comment: While Poisson model is appropriate for this analysis, have you considered Cox model that has better utility for longitudinal data? Was the rate of follow up similar in both groups? If your hypothesis was that care for children with MCA can lead to elevated rates of health care utilization for the mother, regression discontinuity model or interrupted time series may be better tools to test this hypothesis.***

Response: We agree with the Reviewer that a different regression model would be more appropriate when rates are compared. However, the primary outcomes of interest in our study were the period prevalence and mothers’ quantity of health care utilization (primary, inpatient, outpatient, surgical, and psychiatric services). Therefore, we would like to keep the original analytic method.

***Comment: Table 1 bottom: Mortality by strata defined by the number of child hospitalizations in the first year is tricky to interpret. Relatively larger number infants with MCA die soon after birth or before the hospital discharge than infants without MCA. In contrast, no hospitalizations in the first year generally means good health in the average population. I am not sure why these strata (by the number of hospitalizations in the first year) analyses were performed - I would drop them from the paper.***

Response: We included stratified analyses by the number of hospitalizations in the first year to examine a potential dose–response association between the exposure and the outcome. We used the numbers of hospitalization in the first year as a proxy to measure how sick a baby was because it could be predictive of future health care needs of the child, as indicated in the comments. In addition, as indicated by Reviewer #1 in Comment 2, major congenital anomalies cover a wide range of diagnoses, but the actual diagnosis may not account for varied prognoses among individual diagnoses. We believe that the number of hospitalizations in the first year is one of the most reliable ways to assess the severity of MCAs. Contrary to our hypothesis, the mother’s health-care utilization had an inverse relationship with caring for children with multiple hospitalizations. Therefore, we provided two potential explanations: (1) mothers’ tendencies to utilize less health care for their own health and (2) higher mortality rates of children with MCAs led to loss of children, which might attenuate the caregiving burden over time, as well as increase intense grief and profound distress.
